# Pressure‐Triggered Blue Emission of Zero‐Dimensional Organic Bismuth Bromide Perovskite

**DOI:** 10.1002/advs.202004853

**Published:** 2021-02-15

**Authors:** Meng‐En Sun, Ting Geng, Xue Yong, Siyu Lu, Lin Ai, Guanjun Xiao, Jinmeng Cai, Bo Zou, Shuang‐Quan Zang

**Affiliations:** ^1^ Green Catalysis Center and College of Chemistry Zhengzhou University Zhengzhou 450001 P. R. China; ^2^ State Key Laboratory of Superhard Materials College of Physics Jilin University Changchun 130012 P. R. China

**Keywords:** blue luminescence, diamond anvil cell, high pressure, pressure‐induced emission, zero‐dimensional perovskites

## Abstract

Understanding the structure–property relationships in Zero‐dimensional (0D) organic–inorganic metal halide perovskites (**OMHP**s) is essential for their use in optoelectronic applications. Moreover, increasing the emission intensity, particularly for blue emission, is considerably a challenge. Here, intriguing pressure‐induced emission (**PIE**) is successfully achieved from an initially nonluminous 0D **OMHP** [(C_6_H_11_NH_3_)_4_BiBr_6_]Br·CH_3_CN (**Cy_4_BiBr_7_**) upon compression. The emission intensity increases significantly, even reaching high‐efficiency blue luminescence, as the external pressure is increased to 4.9 GPa. Analyses of the *in situ* high‐pressure experiments and first‐principle calculations indicate that the observed PIE can be attributed to the enhanced exciton binding energy associated with [BiBr_6_]^3–^ octahedron distortion under pressure. This study of **Cy_4_BiBr_7_** sheds light on the relationship between the structure and optical properties of **OMHP**s. The results may improve potential applications of such materials in the fields of pressure sensing and trademark security.

## Graphical abstract

The deformation of bismuth bromide octahedra stimulated by lattice shrinkage enhances exciton–phonon coupling, resulting in bright blue emission at pressure of 4.9 GPa or low temperature for the Zero‐dimensional (0D) organic–inorganic metal halide perovskite [(C_6_H_11_NH_3_)_4_BiBr_6_]Br·CH_3_CN (**Cy_4_BiBr_7_**). This research provides a new research approach for the acquisition of the high efficiency blue emitting materials.

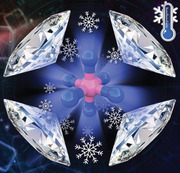

## 1 Introduction

Organic–inorganic metal halide perovskites (**OMHP**s) have attracted considerable attention due to their exceptional properties, such as excellent charge transport, exceptional band gap tenability, and efficient luminescence.^[^ ^1^ ^]^ For Zero‐dimensional (0D) **OMHP**s, individual metal halide octahedral anions are completely surrounded and isolated by organic cations.^[^ ^2^ ^]^ Because of the strong spatial confinement of isolated metal halides, the emissions of 0D **OMHP**s feature broadband spectra, large Stokes shifts, and high photoluminescence (PL) quantum efficiencies.^[^ ^3^ ^]^ These remarkable features make them excellent candidates for use as solid‐state phosphors. Therefore, the recent studies have favored 0D **OMHP**s.^[^ ^4^ ^]^ However, a large Stokes shift is the main reason for the lack of blue luminescence in 0D **OMHP**s. Thus, developing an effective strategy to achieve high‐efficiency blue luminescence with 0D **OMHP**s remains a pressing challenge.

Pressure, as an independent thermodynamic parameter, has been the focus of achieving efficient blue luminescence for 0D **OMHP**s. Since the influence of composition changes is avoided, hydrostatic pressure is considered to be the easiest way to adjust the physical properties of materials by changing the electronic structure.^[^ ^5^ ^]^ Recently, there have been some reports about pressure enhancing the emission of **OMHP**s.^[^ ^6^ ^]^ However, due to the structural distortion of metal halides under pressure, the self‐trapping exciton binding is enhanced, which usually leads to a red‐shift for emission peak (i.e., the Stokes shift increases). In particular, Zhang et al. found that when (C_6_H_5_C_2_H_4_NH_3_)_2_PbBr_4_ was subjected to enhanced pressure, the blue emission of the free‐exciton state weakened or disappeared, and the red emission of the self‐trapping exciton state appeared or increased.^[^ ^7^ ^]^ Therefore, overcoming large structural distortion under pressure is a considerable challenge.

Consequently, decreasing the Stokes shift is the key to achieving blue luminescence for 0D **OMHP**s. It has been reported that heavy ns^2^ electron structure atoms (such as lead and bismuth) cause a small Stokes shift due to the strong spin–orbit coupling perturbations in metal halide crystals.^[^ ^8^ ^]^ However, the use of lead ions may restrict large‐scale manufacturing due to its toxicity and possible bioaccumulation.^[^ ^9^ ^]^ Bi^3+^ has the same 6S^2^6P^0^ electronic structure as Pb^2+^ and a similar ionic radius, and Bi‐based halide perovskites exhibit lower toxicity and better chemical stability than lead.^[^ ^10^ ^]^ Thus, Bi^3+^ is an excellent candidate for use in lead‐free low‐toxicity perovskite materials.^[^ ^11^ ^]^ In addition, rigidifying the chemical environment around metal halide anions in crystals is also an efficient method for reducing the Stokes shift of 0D organic–inorganic hybrid materials.^[^ ^12^ ^]^

Herein, we perform a systematic high‐pressure study of a 0D **OMHP**s [(C_6_H_11_NH_3_)_4_BiBr_6_]Br·CH_3_CN (**Cy_4_BiBr_7_**). The organic cation C_6_H_11_NH_3_^+^ confines the bismuth bromide octahedron in a relatively rigid hydrogen bond network. **Cy_4_BiBr_7_** exhibits an unexpected pressure‐induced emission (**PIE**) at room temperature (RT) when the intrinsically nonemitting crystals are compressed to 4.9 GPa. The pressure‐dependent absorption spectra, angle‐dispersive synchrotron X‐ray diffraction (ADXRD) patterns, and Raman spectra combined with temperature‐dependent emissions and single‐crystal X‐ray diffraction (SCXRD) results, and theoretical calculations indicate that the attracting blue emission resulted from the exciton transitions with the enhanced exciton binding energy that are triggered by the minor distortion of the [BiBr_6_]^3–^ octahedron under pressure. The results demonstrate that lead‐free metal halides are promising for high‐efficiency blue luminescence and that pressure‐controlled emission can overcome the limits of conventional synthetic chemistry.

## 2 Results and Discussion

**Figure 1:**
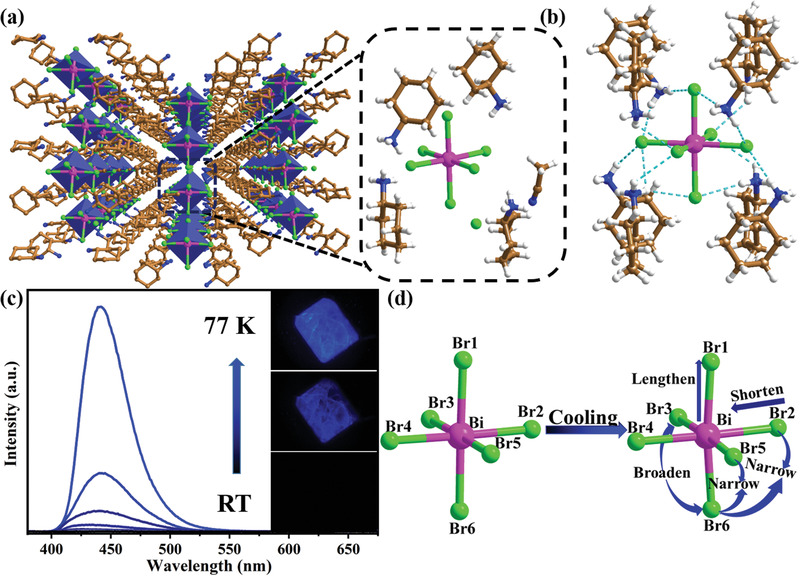
a) View of the structure of**Cy_4_BiBr_7_** at RT. b) View of N—H···Br hydrogen bonding interactions (turquoise dashed line) between an individual [BiBr_6_]^3–^ and C_6_H_11_NH_3_^+^ at RT. Color codes: pink = Bi; green = Br; brown = C; blue = N; white = H; blue polyhedral = BiBr_6_. c) Temperature‐dependent PL spectra of **Cy_4_BiBr_7_** from RT down to 77 K in the solid state under ambient conditions. d) Schematic of the temperature stimulation and distortion of the [BiBr_6_]^3–^ octahedron in **Cy_4_BiBr_7_** under ambient conditions.

The precise structure of**Cy_4_BiBr_7_** was determined by SCXRD (Tables S1 and S2, Supporting Information). As depicted in **Figure** **1**a, the Bi^3+^ cation occupies the center of the octahedron, and six bromide ions are located at the vertices. The [BiBr_6_]^3–^ octahedra are periodically embedded in the crystal lattice together with the organic cations C_6_H_11_NH_3_^+^ to from bulk materials, thus creating an 0D **OMHP** structure at the molecular level. Hydrogen bond interactions among the various components are not neglected in **Cy_4_BiBr_7_** (Tables S3 and S4, Supporting Information). As shown in Figure 1b, [BiBr_6_]^3–^ octahedra connect to the surrounding organic cation C_6_H_11_NH_3_^+^ through strong N‐H···Br hydrogen bonds. Acetonitrile molecules are also linked by hydrogen bonds with the [BiBr_6_]^3–^ octahedron (Figure S1, Supporting Information). The purity of Cy_4_BiBr_7_ was confirmed by the well‐overlapped powder X‐ray diffraction (PXRD) patterns between the as‐synthesized samples and the simulated single‐crystal data (Figure S2, Supporting Information).

**Figure 2:**
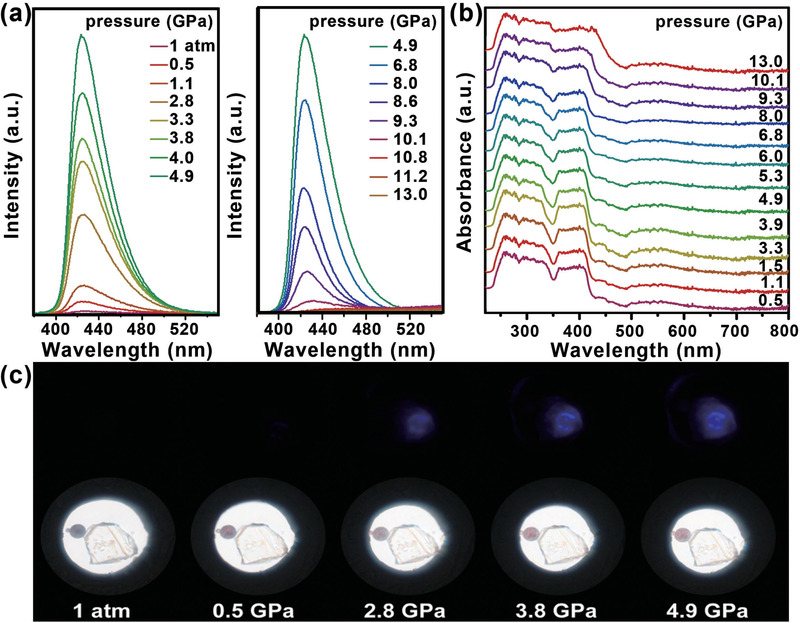
a) Pressure‐dependent PL spectra of **Cy_4_BiBr_7_**. b) Changes in the absorption spectra of **Cy_4_BiBr_7_** with increasing pressure. c) Corresponding PL photographs under UV irradiation (*λ*_ex_ = 355 nm) and *in situ* high‐pressure optical micrographs in a diamond anvil cell at selected pressures of 1 atm and 0.5, 2.8, 3.8, and 4.9 GPa.

Unexpectedly, **Cy_4_BiBr_7_** initially did not exhibit a PL response to external pressure and showed bright blue emission as the pressure increased. As shown in **Figure** **2**a, with increasing pressure, an emission peak suddenly appears. With a further increase in pressure, the PL of **Cy_4_BiBr_7_** exhibits an apparent enhancement. Note that the corresponding PL intensity shows a remarkable increase with increasing pressure, eventually reaching a maximum at a pressure of 4.9 GPa. Upon further compression, the PL intensity gradually decreased and was reached when the pressure approached 13.0 GPa. This interesting **PIE** phenomenon can also be demonstrated from the changes in the PL photograph under laser irradiation at 355 nm. It is obvious that when the pressure increases to a certain value, the pressure‐dependent PL photographs clearly turn bright, as shown in Figure 2c. Similarly, the **Cy_4_BiBr_7_** crystals also gradually display blue PL as the temperature decreases under ambient conditions (Figure 1c). This blue emission peak at *λ*_EM_ = 441 nm has a small Stokes shift of 47 nm and remains unchanged within a large excitation wavelength range (Figures S3–S6, Supporting Information).

The absorption spectra of **Cy_4_BiBr_7_** upon compression are shown in Figure 2b. The **Cy_4_BiBr_7_** crystals are colorless under ambient light and ambient conditions and have an absorption edge located at ≈400 nm (Figure S7, Supporting Information). It is found that the absorption spectra of Cy_4_BiBr_7_ are greatly stable up to 13.0 GPa. With the increase in external pressure, the absorption spectra underwent a successive redshift, indicating the band gap narrowing of **Cy_4_BiBr_7_** with the pressure changes. This band gap engineering as a function of pressure should be highly related to the octahedral motif changes with increasing pressure.

**Figure 3:**
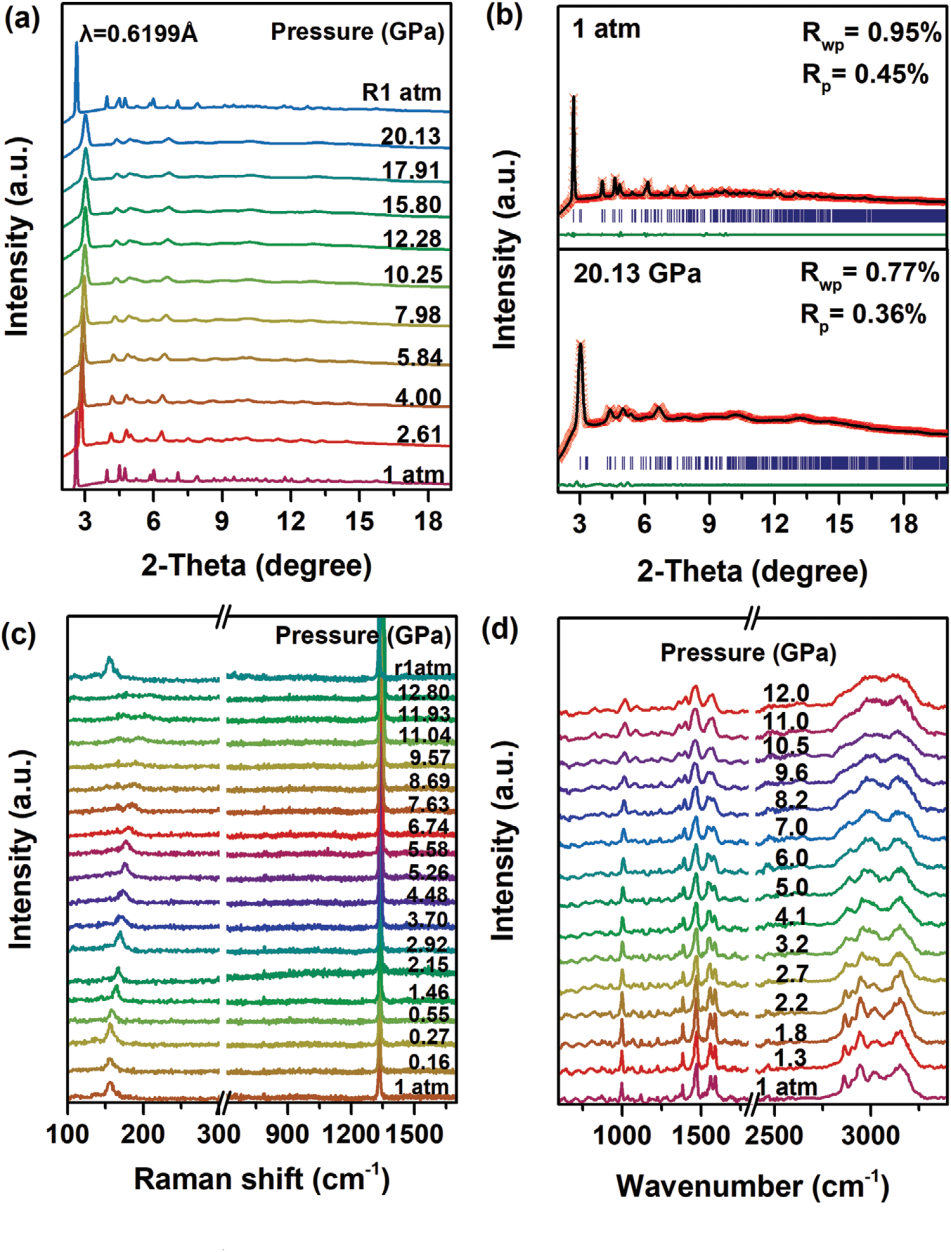
a) Representative *in situ* ADXRD patterns of **Cy_4_BiBr_7_** in the presence of silicon oil as the pressure‐transmitting medium during the high‐pressure experiments. b) Rietveld refinements of **Cy_4_BiBr_7_** at ambient conditions and 20.13 GPa. c) Raman spectra of **Cy_4_BiBr_7_** at high pressure. d) Pressure‐dependent mid‐infrared spectra of **Cy_4_BiBr_7_**.

To investigate the relationship between the optical properties and structural evolution upon compression, we performed *in situ* high‐pressure ADXRD up to ≈20.13 GPa. The evolutionary information for the structure of **Cy_4_BiBr_7_** upon compression is shown in **Figure** **3**a. The first peak centered around 2*θ* = 2.6° corresponding to the Bragg (001) reflection, and the others have labeled shown in Figure S8, Supporting Information. As the pressure increased, all Bragg diffraction peaks monotonically shifted toward higher diffraction angles, which resulted from the pressure‐induced lattice contraction. Moreover, no new peaks appeared upon compression, indicating that **Cy_4_BiBr_7_** did not experience any structural phase transition. And ADXRD results analysis also revealed that**Cy_4_BiBr_7_** possesses the same space symmetry at ambient conditions and 20.13 GPa (Figure 3b). The Raman spectra of **Cy_4_BiBr_7_** with a frequency distribution from 100 to 1700 cm^−1^ upon compression are shown in Figure 3c. Under ambient pressure, the vibrational mode (155.78 cm^–1^) was associated with the [BiBr_6_]^3–^ octahedra, and this movement originated from the distortion of the [BiBr_6_]^3–^ octahedron (Figure S9, Supporting Information).^[^ ^13^ ^]^ As the pressure increased, the vibrational mode of A_lg_ in [BiBr_6_]^3–^ octahedra showed a normal movement in the direction of a high Raman shift, which was attributed to the reduction of interatomic distance.^[^ ^14^ ^]^ As the progressive deteriorated crystallinity under higher pressure, the vibrational intensity of A_1g_ mode weakened until disappeared. The peak around 1334.71 cm^–1^ at ambient pressure can be attributed to the vibration modes of C_6_H_11_NH_3_^+^ cation.^[^ ^15^ ^]^ With increasing pressure, the vibrational modes of C_6_H_11_– show a monotonic blue‐shift. However, the vibrational modes of –NH_3_^+^ hardly shifted with pressure (Figure S10, Supporting Information). We speculate that this was due to the pressure‐induced lattice shrinkage limiting the atomic vibration and concurrently increasing the interreaction between the C_6_H_11_NH_3_^+^ cation and the [BiBr_6_]^3–^ anion. It was worthy to note that the ADXRD and Raman peaks returned to the initial positions upon the complete release of pressure, indicating the pressure‐dependent reversibility of**Cy_4_BiBr_7_**.

To further investigate the behavior of the organic and its interplay with the inorganic moiety, we carried out high‐pressure infrared absorption spectrum (IR) experiments at RT to provide information about the C_6_H_11_NH_3_^+^ cation changes, as shown in Figure 3d. As pressure rises, vibrational peaks from the organic moiety shift and broaden, which can be exactly elucidated by the intensified interatomic force and the decreased crystallinity.^[^ ^16^ ^]^ Both blue‐shifts and red‐shifts were observed in characteristic modes (Figures S11 and S12, Supporting Information). The volume of the lattice underwent considerable shrinkage, leading to strenuous repulsion between the [BiBr_6_]^3–^ octahedron and the electron waves on the H atoms in the cyclohexyl group. Hence, the frequency of C—H stretching in C_6_H_11_– increased. However, both the N—H stretching and bending modes in the NH_3_^+^ experienced red‐shift as pressure increased. This reverse change trend for N—H modes relative to the C—H and C—N modes can be attributed to the cationic charge on NH_3_^+^. The pressure‐induced lattice shrinkage further enhanced the electrostatic attraction between the cationic charge in NH_3_^+^ and their neighboring anionic charge on the [BiBr_6_]^3–^ anion.

**Figure 4:**
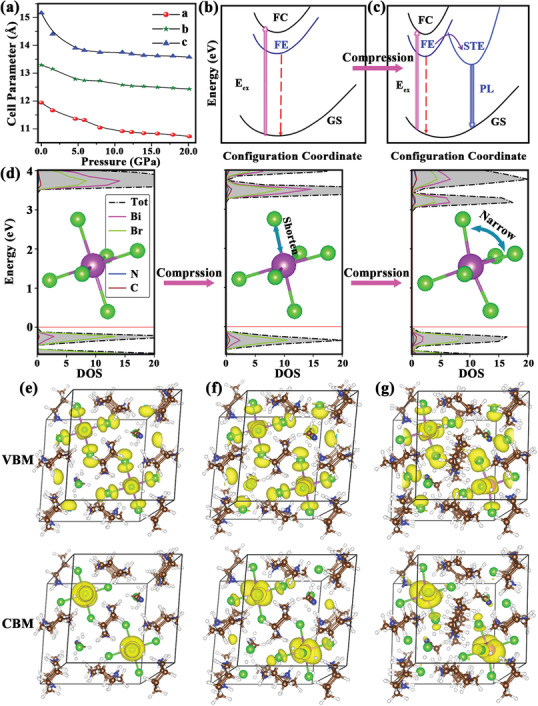
a) The lattice constants of **Cy_4_BiBr_7_** as a function of pressure. b,c) Illustrations of the **PIE** mechanism associated with free excitons under ambient conditions and upon compression. Ground state (GS), free exciton (FE) state, self‐trapped exciton (STE) state, exciton transition energy (*E*_ex_), and free‐carrier (FC) state. d) Computed DOS at 0, 5.0, and 15.0 GPa. The inserts describe the changes in the local [BiBr_6_]^3–^ octahedron upon compression: shortening of Bi—Br bonds and narrowing of Br—Bi—Br angles. The computed VBM and CBM are ambient conditions at e) 0 GPa, f) 5.0 GPa, and g) 15.0 GPa. The main components are the d orbitals of Bi and p orbitals of Br.

Rietveld refinements of the ADXRD patterns can obtain the unit cell parameters of**Cy_4_BiBr_7_** at different pressures (Table S5, Supporting Information). The variations in cell parameters and cell volume of **Cy_4_BiBr_7_** upon compression are displayed in **Figure** **4**a and Figure S13, Supporting Information. It is obvious that the unit cell parameters and cell volume of **Cy_4_BiBr_7_** were greatly reduced below 5.0 GPa. Above 5.0 GPa, the rate of reduction slows down. Notably, cell parameters and cell volume of**Cy_4_BiBr_7_** were continuously compressed without collapses, which confirmed that **Cy_4_BiBr_7_** did not undergo phase transition within the detection pressure range. More importantly, such structural evolutions correspond to the trend in the experimental measurements of emission enhancement.

Through the above analysis, we can guess that there was relevance between the **PIE** of **Cy_4_BiBr_7_** and the lattice shrinkage. Moreover, the low‐temperature SCXRD data under ambient conditions verified that lattice shrinkage induced the blue emission of **Cy_4_BiBr_7_** (Figure S14, Supporting Information). As shown in Figure 1d, upon decreasing the temperature, the bond lengths and bond angles of the [BiBr_6_]^3–^ octahedron changed with shrinkage of the lattice. The larger values of ∆*d* (Bi—Br bond length distortion) and *δ* ^2^ (BiBr_6_ octahedral angle variance) at low temperatures than RT intuitively reflected this change (see Supporting Information for details).^[^ ^17^ ^]^ We found that the bismuth bromide octahedron in **Cy_4_BiBr_7_** has a lesser structural distortion than other 0D **OMHP**s (Table S6, Supporting Information). This may be used to explain the small Stokes shift blue emission of **Cy_4_BiBr_7_** at low temperature or high pressure. It should be noted that there was also no phase change between low temperatures and RT (Figure S15, Supporting Information). In metal halide perovskites, the distortion of the metal halide octahedron can improve exciton–phonon coupling, thereby activating PL.^[^ ^18^ ^]^ Thus, the abovementioned PIE of **Cy_4_BiBr_7_** at RT should originate from the slight distortion of the [BiBr_6_]^3–^ octahedron upon compression.

Density functional theory (DFT) calculations were performed to investigate the structural, electronic, and spectral properties of **Cy_4_BiBr_7_**. The [BiBr_6_]^3–^ octahedra were separated to form 0D structure (Figure 4d) that could be altered under pressure. Upon compression to 5.0 GPa, the Bi—Br bond lengths were shortened from ≈2.80 Å under ambient conditions to 2.78 Å, and the Br—Bi—Br bond angles were narrowed by 4°. Upon further compression to 15.0 GPa, the Bi—Br bond length became even shorter at ≈2.70 Å, and the Br‐Bi‐Br angles decreased by 10° (Figure 4d). This analysis indicates that the observed **PIE** is from the local structural changes in the [BiBr_6_]^3–^ octahedron. The electronic properties evolved with structural changes. The density of states (DOS, Figure 4d) of **Cy_4_BiBr_7_** around the Fermi level mainly included the *d* orbitals of Bi atoms and *p* orbitals of Br atoms. Under ambient conditions, the valence band maximum (VBM) mainly encompassed the *d* orbitals of Bi and *p* orbitals of Br, and the conduction band minimum (CBM) was contributed by the *d* orbitals of Bi and *p* orbitals of the two Br atoms in each [BiBr_6_]^3–^ octahedron (Figure 4e). Upon compression, the Bi—Br bond interactions strengthened and displayed large overlap at high pressures (Figure 4f,g). Another important feature is that the *d* orbitals of Bi that form CBM are relatively diffuse, and the majority of Br atoms in the [BiBr_6_]^3–^ octahedron contribute to the CBM. Thus CBM upon compression in general shows better overlaps than under ambient conditions. Hence, the VBM moves upward and the CBM moves downward to the Fermi level under compression, and the band gaps gradually decrease (Figure 4d).

Based on the above results, we propose that the changes in the electronic properties are responsible for the **PIE** of **Cy_4_BiBr_7_**. Incident photons are absorbed by promoting electrons from the GS into higher energy excited states, including the FC state and FE state (Figure 4b). Under ambient conditions, the Bi—Br bonds are weak, and the lattice is characterized by weak electron–phonon coupling, which results in the bound excitons being readily detrapped from the STE state to the FE state by thermal activation, thus hindering emission. However, upon compression, the large CBM within the distorted octahedral motif can accommodate the STE state, and the strong Bi—Br bonds enhance electron–phonon coupling. Stable STE states are thus formed and enhance the emission intensity (Figure 4c).

## 3 Conclusion

In conclusion, we have designed and synthesized a **Cy_4_BiBr_7_** crystal with a lead‐free 0D **OMHP** structure. Notably, by applying mild pressures, we were able to make originally nonluminescent crystals emit a bright blue color. Specifically, the pressure deformed the bismuth bromide octahedron, resulting in enhanced electron–phonon coupling. *In situ* high‐pressure ADXRD and Raman spectra, as well as the DFT results, further corroborate this **PIE** mechanism. Furthermore, the variable‐temperature SCXRD analysis supported this finding. Our work verifies the possibility of PL engineering in 0D lead‐free **OMHP**s with enhanced functionality through high‐pressure structural modulation. Additionally, our study provides a new avenue for exploiting high‐efficiency blue luminescence materials.

## Conflict of Interest

The authors declare no conflict of interest.

## Supporting information

Supporting Information

## Data Availability

Research data are not shared.
