# Supplementary material for: Pressure‐Triggered Blue Emission of Zero‐Dimensional Organic Bismuth Bromide Perovskite
Source: Adv Sci (Weinh). 2021 Feb 15;8(9):2004853. doi: 10.1002/advs.202004853 (PMC8097370; doi:10.1002/advs.202004853)
Supplement: Supplementary file 1 — Supporting Information [file ADVS-8-2004853-s001.pdf]

© 2021 Wiley-VCH GmbH

ADVANCED SCIENCE
Open Access

Supporting Information

for *Adv. Sci.*, DOI: 10.1002/advs.202004853

Pressure-triggered Blue Emission of Zero-Dimensional Organic Bismuth Bromide Perovskite

Meng-En Sun,[†] Ting Geng,[†] Xue Yong, Siyu Lu, Lin Ai, Guanjun Xiao,* Jinmeng Cai, Bo Zou,* Shuang-Quan Zang**

Supporting Information

Pressure-triggered Blue Emission of Zero-Dimensional Organic Bismuth Bromide Perovskite

Meng-En Sun,[†] *Ting Geng,*[†] *Xue Yong, Siyu Lu,* Lin Ai, Guanjun Xiao,* Jinmeng Cai, Bo Zou,* Shuang-Quan Zang**

Experimental Section

Materials. All reagents and solvents used were of commercially available reagent grade and were used without any additional purification.

Synthesis of [($C_6H_{11}NH_3$)$_4$BiBr$_6$]Br·CH_3CN (Cy$_4$BiBr$_7$). Cyclohexylammonium bromide (320 mg, 0.56 mmol), Bi_2O_3 (300 mg, 0.86 mmol), HBr aqueous solution (40%, 2 mL) and CH_3CN (30 mL) were mixed in a beaker. The resulting olive-colored solution was allowed to evaporate slowly in the dark at RT for a week to obtain bulk colorless crystals of **Cy$_4$BiBr$_7$**.

Single-crystal X-ray diffraction analysis (SCXRD). SCXRD measurements of **Cy$_4$BiBr$_7$** were performed on a Rigaku XtaLAB Pro diffractometer with Cu-Kα radiation (λ = 1.54178 Å) at RT and 100 K. Data collection and reduction were performed using the program CrysAlisPro [S1]. The two structures were all assessed with direct methods (SHELXS) [S2] and refined by full-matrix least squares in F2 using OLEX2, [S3] which utilizes the SHELXL-2015 module. [S4] The crystal structures were visualized in DIAMOND 3.2. [S5]

Powder X-ray Diffraction (PXRD). The PXRD patterns of the samples were recorded on a D/MAX-3D diffractometer (Cu Kα, λ = 1.54178 Å). Simulated powder patterns were obtained with Mercury software and the crystallographic information file (CIF) from a single-crystal X-ray experiment.

Thermogravimetric (TG) analysis. TG analyses of **Cy$_4$BiBr$_7$** were performed with a TA Q50 thermal analyzer from RT to 400 °C at a heating rate of 10 °C min^{-1} under a nitrogen atmosphere.

Differential scanning calorimeter (DSC). DSC analyses of **Cy$_4$BiBr$_7$** were performed on a DSC Q25 thermal analyzer from 40 °C to -140 °C with heating to 40 °C at a rate of 10 °C min^{-1} under a nitrogen atmosphere.

UV-visible absorption spectra. UV-visible absorption spectra were recorded using a Hitachi UH4150 spectrophotometer (Integrating Sphere) in the range of 240-800 nm.

Luminescence measurements. Solid-state emission and excitation spectra at different temperatures were recorded with a HORIBA FluoroLog-3 fluorescence spectrometer, and luminescence microscopy images were recorded with an Olympus BX53 microscope. Luminescence decay was measured with the HORIBA FluoroLog-3 fluorescence spectrometer equipped with a 355 nm laser operating in time-correlated single-photon counting mode (TCSPC) with a resolution time of 11 ms.

Computational details. The electronic structure calculations were performed using the Vienna Ab initio Simulation Package (VASP) code and soft projector-augmented wave (PAW) pseudopotentials. The Perdew-Burke-Ernzerhof (PBE) exchange-correlation

functional was used. Electron orbitals were expanded by plane wave basis sets with an energy cut-off of 450 eV. To sample the Brillouin zone, we used a 4×4×4 *k*-point mesh for geometry optimization and a 4×4×4 mesh for the density state calculations. The force on any atom converged to less than 0.01 eV/Å, and the self-consistency of the total energy for each ionic step was set to 1×10^{-6} eV.

Analysis of octahedral distortion. The degree of octahedral distortions was determined by using the following parameters: [S6, S7]

$$\text{Bond length distortion: } \Delta d = \frac{1}{6}\sum_{i=1}^{6}[(d_{\mathrm{i}} - d_0)d_0]^2$$

$$\text{Octahedral angle variance: } \delta^2 = \frac{1}{11}\sum_{i=1}^{12}(\theta_i - 90)^2$$

where d_0 is the average Bi-Br bond length, d_i represents the individual Bi-Br bond lengths, and θ_i represents the individual Br-Bi-Br bond angles of the octahedron. The Δd and δ^2 values at RT are $2.76*10^{-6}$ and 0.61, respectively, and they increase to $2.45*10^{-5}$ and 5.62 at 100 K.

High pressure generation. High-pressure experiments were carried out with a symmetric diamond anvil cell (DAC). The sample and a small ruby ball were loaded into the 150 μm-diameter chamber of a DAC, constructed from a T301 steel gasket pre-indented to a thickness of 45 μm. A small ruby ball was load into the sample chamber along with the **Cy_4BiBr_7** samples for pressure calibration using the ruby fluorescence method. In high-pressure experiments, silicon oil was utilized as the pressure transmitting medium (PTM) for optical absorption, PL and ADXRD experiments. The PTM did not have any detectable effect on the behavior of **Cy_4BiBr_7** under pressure. All of the measurements were performed at room temperature.

***In situ* high pressure PL, UV-visible absorption, Raman, infrared absorption and XRD measurements.** PL spectra were measured by using a 355 nm laser excitation line at 10 mW. UV-visible absorption spectra were measured by using a deuterium-halogen light source, and the measurements between 240 nm and 800 nm. In PL and UV-visible absorption experiments, the fiber spectrometer was an Ocean Optics QE6500 spectrometer. Raman spectra were collected by a Raman spectrometer (iHR 550, Syncerity, Horiba Jobin Yvon) with a 785 nm laser excitation line at 10 mW. PL micrographs of the samples were obtained using a camera (Canon Eos 5D mark II) equipped on a microscope (Ecilipse TI-U, Nikon). In high pressure infrared absorption measurements were carried out using Nicolet iN10 FT-IR Spectrometer (Thermo Fisher Scientific). The camera can record the photographs under the same conditions including exposure time and intensity. We performed in situ high pressure angle-dispersive XRD measurements with wavelengths of 0.6199 Å at beamline BL15U1, Shanghai

Synchrotron Radiation Facility (SSRF), China. We used CeO_2 as a standard sample to do the calibration. The two-dimensional Debye-Scherrer diffraction rings were recorded using an imaging plate detector and integrated into the one-dimensional profile using Fit2D program. Structure refinements were carried out using Materials Studio program. All of the high-pressure experiments were conducted at room temperature.

Figures and tables

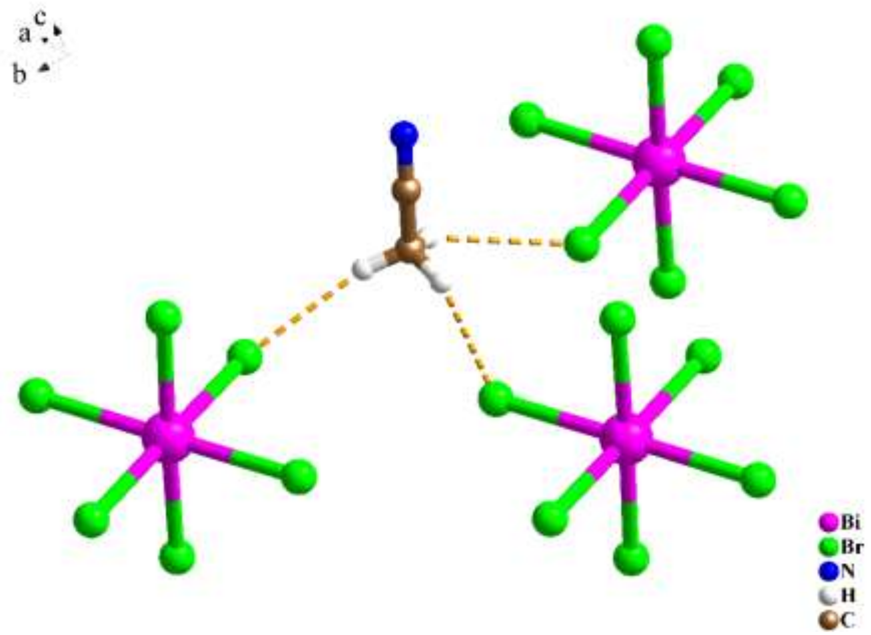

Figure S1. View of the C–H···Br (orange dashed line) hydrogen bonding interaction between an individual acetonitrile molecule and the neighboring $[BiBr_6]^{3-}$ in **Cy_4BiBr_7** at RT.

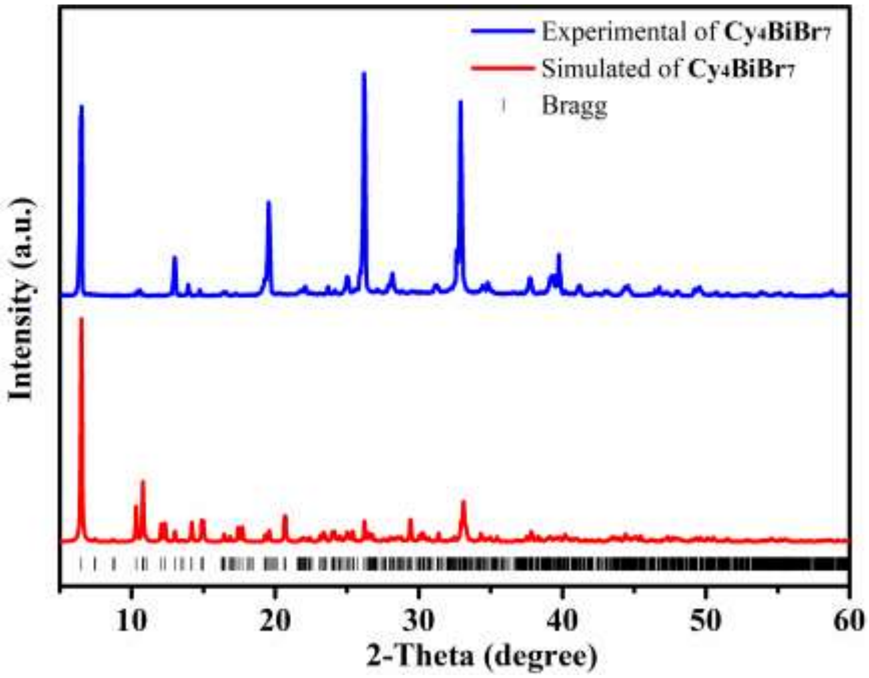

Figure S2. PXRD patterns of **Cy_4BiBr_7** crystal powders.

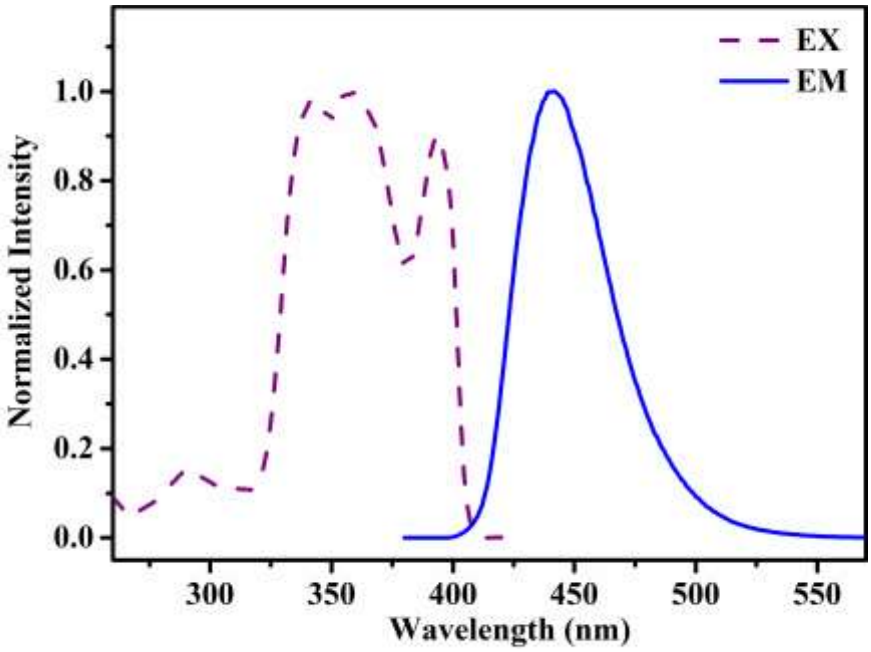

Figure S3. Excitation spectra (monitored at 441 nm) (dotted line) and emission spectra (excited at 358 nm) (solid line) of solid-state **Cy_4BiBr_7** at 77 K. EX = excitation, EM = emission. The intensity of peaks was normalized.

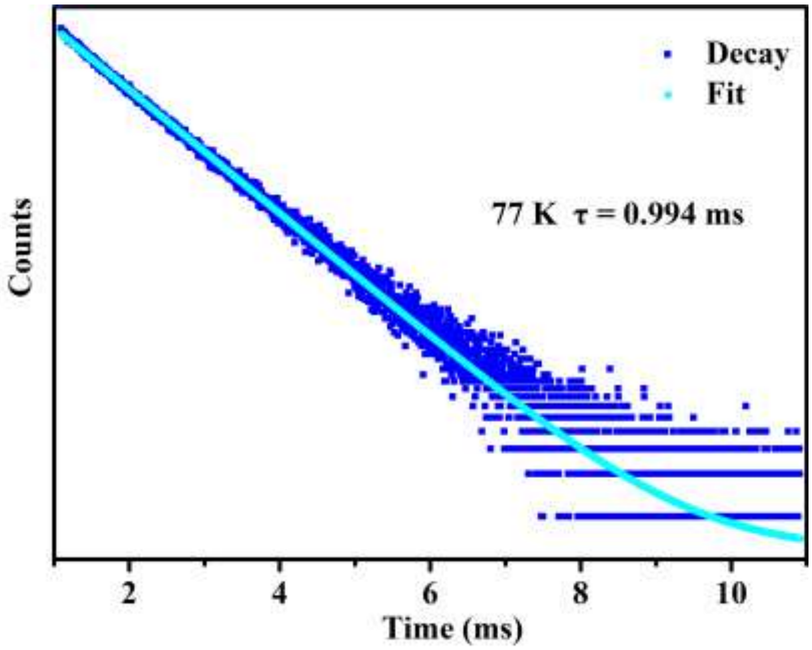

Figure S4. Representative time-resolved photoluminescence decays (λ_{EX} = 441 nm) of solid-state **Cy_4BiBr_7** at 77 K.

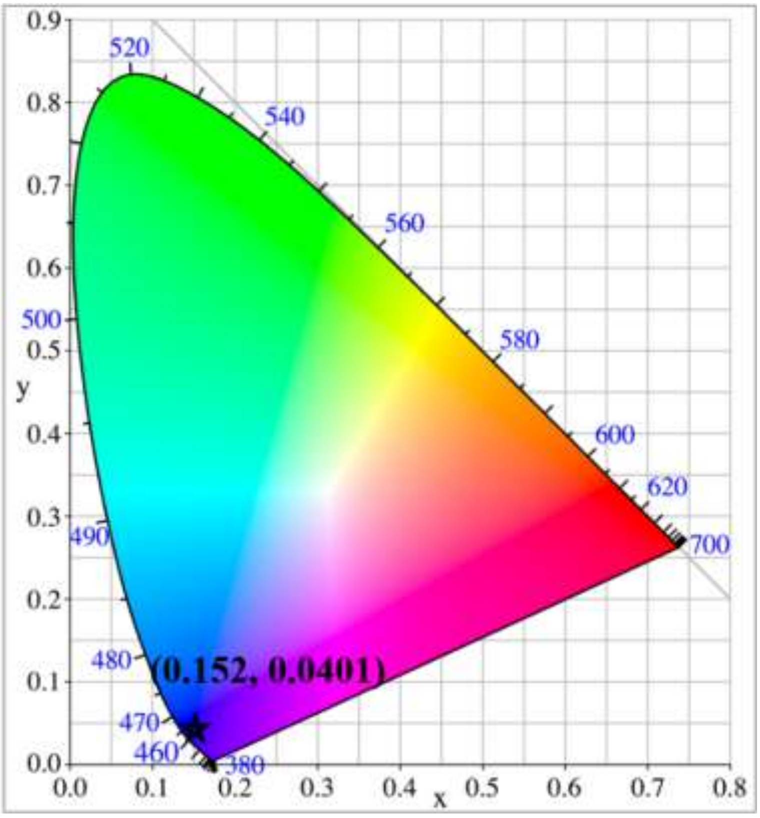

Figure S5. Commission Internationale de l'Eclairage (CIE) chromaticity coordinates for **Cy_4BiBr_7** at 77 K.

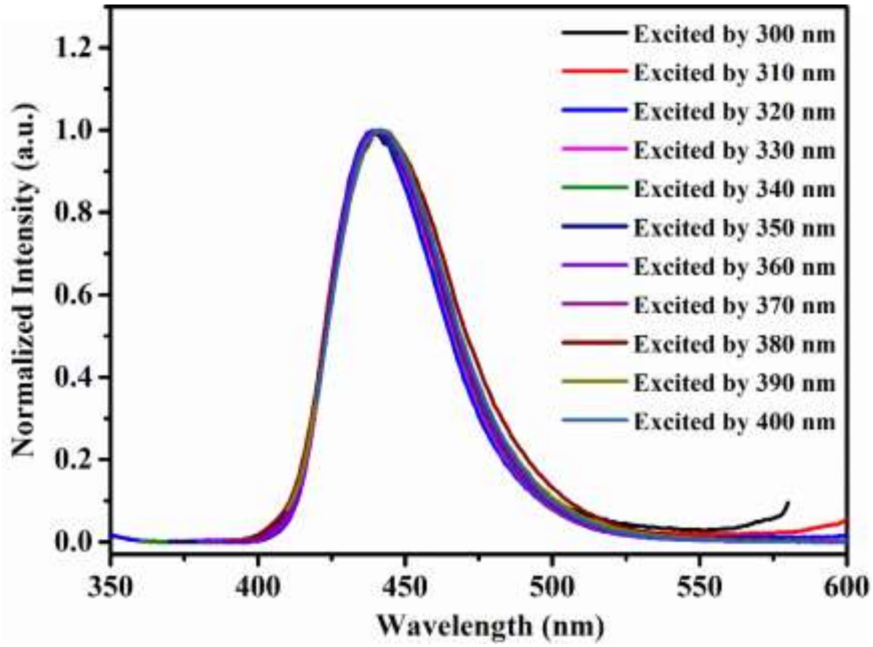

Figure S6. Emission spectra of **Cy_4BiBr_7** excited based on wavelengths from 300 nm to 400 nm with an interval of 10 nm at 77 K.

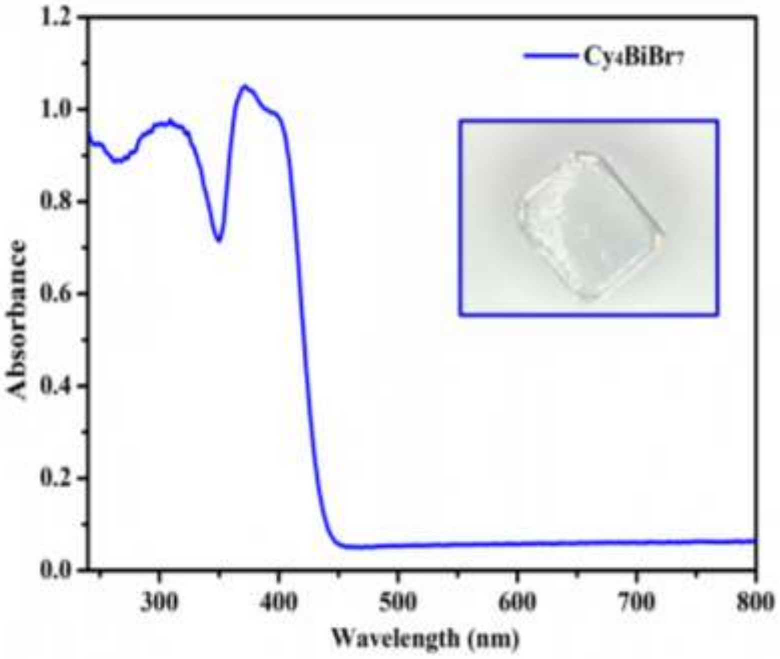

Figure S7. UV-vis diffuse reflectance spectra of solid-state **Cy_4BiBr_7** under ambient conditions. Inset: a crystal photo of **Cy_4BiBr_7** under ambient light.

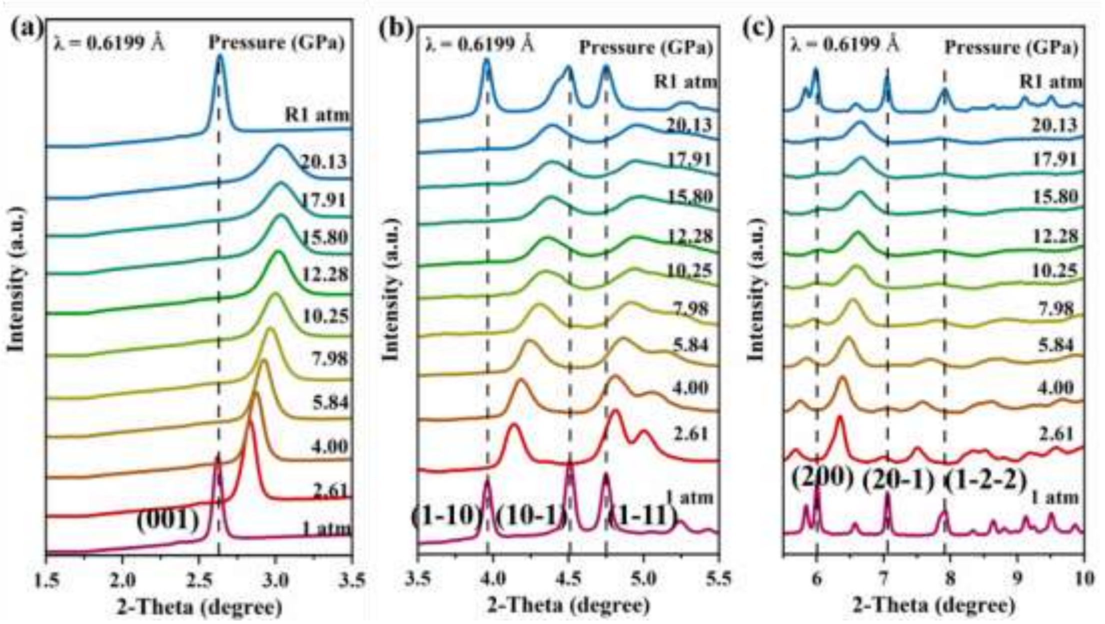

Figure S8. Selected ADXRD patterns of **Cy_4BiBr_7**.

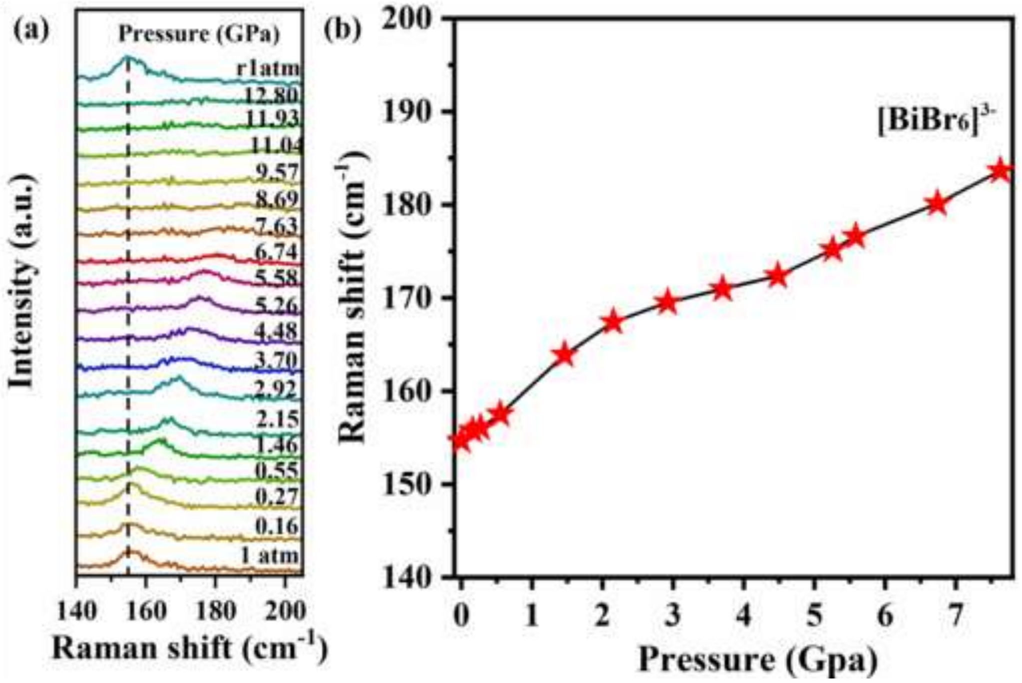

Figure S9. (a) Raman patterns of $[BiBr_6]^{3-}$ octahedra upon compression. (b) Raman shift of $[BiBr_6]^{3-}$ octahedra as a function of pressure.

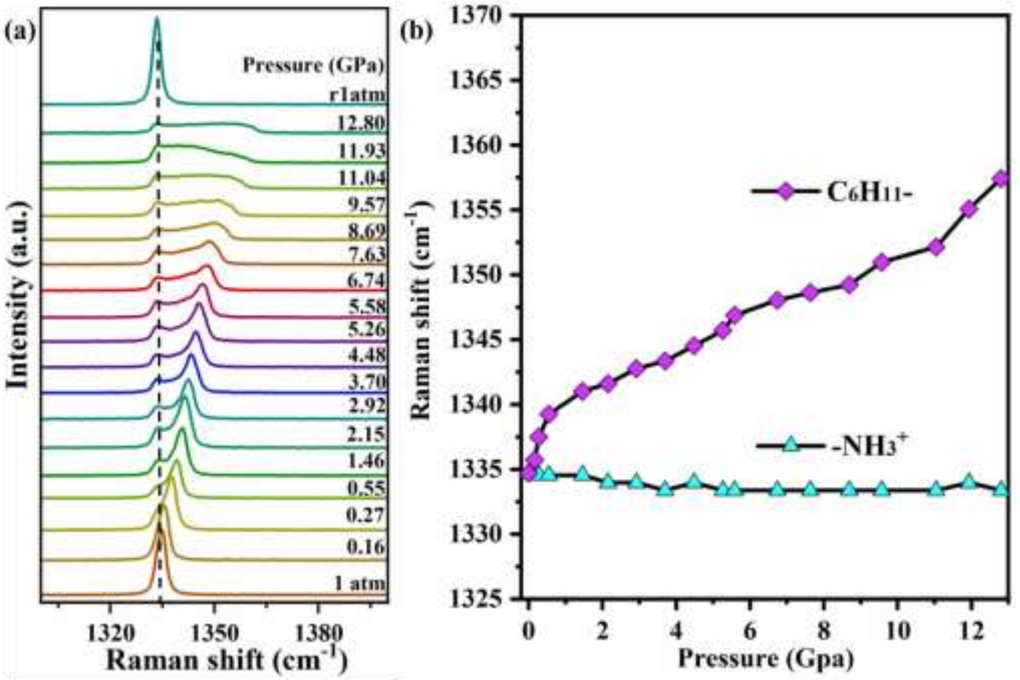

Figure S10. (a) Raman patterns of $C_6H_{11}NH_3^+$ cation upon compression. (b) Raman shift of $C_6H_{11}NH_3^+$ cation as a function of pressure.

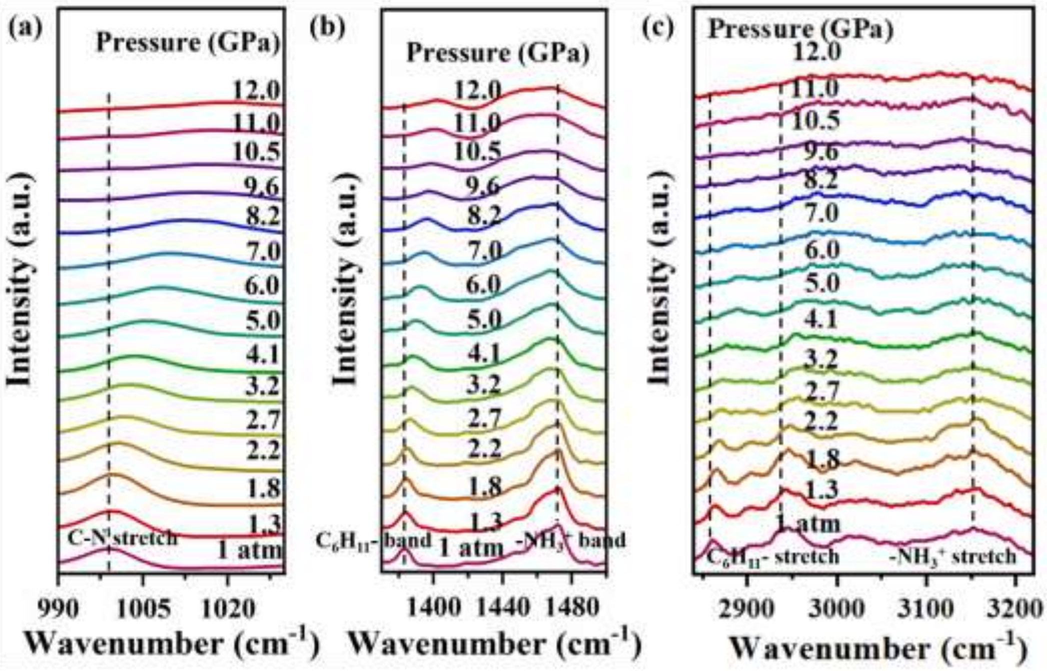

Figure S11. Selected IR spectra patterns of $\mathbf{Cy_4BiBr_7}$.

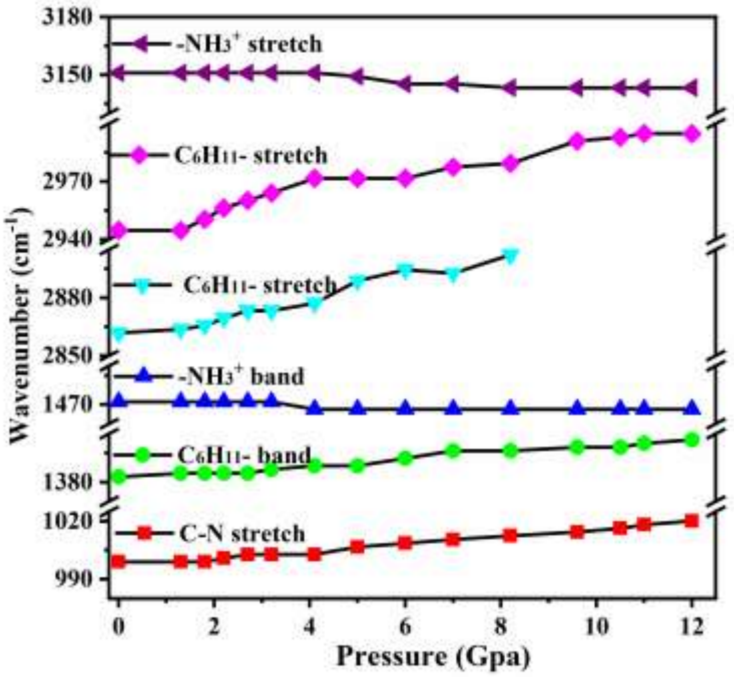

Figure S12. IR shift of $\mathbf{Cy_4BiBr_7}$ as a function of pressure.

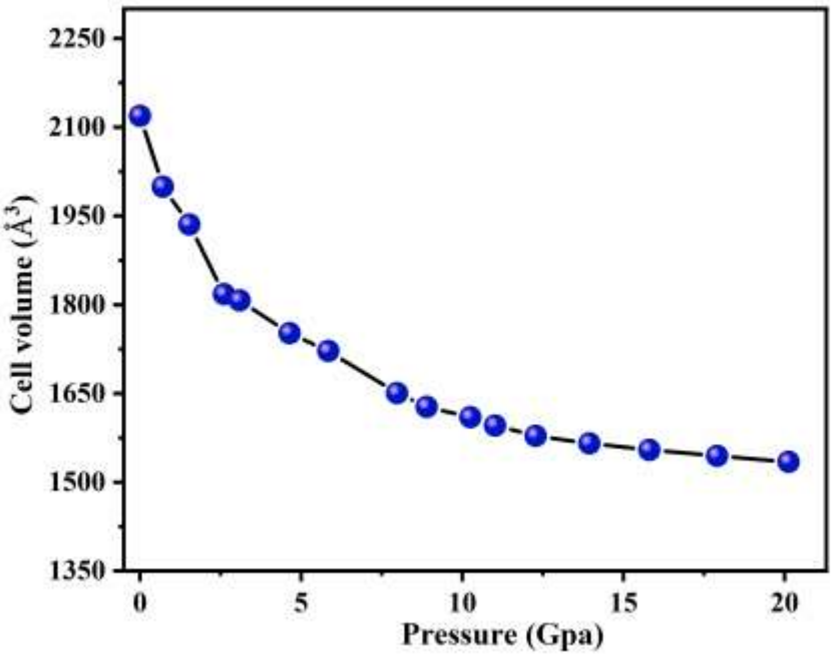

Figure S13. Cell volume of $\mathbf{Cy_4BiBr_7}$ at different pressures.

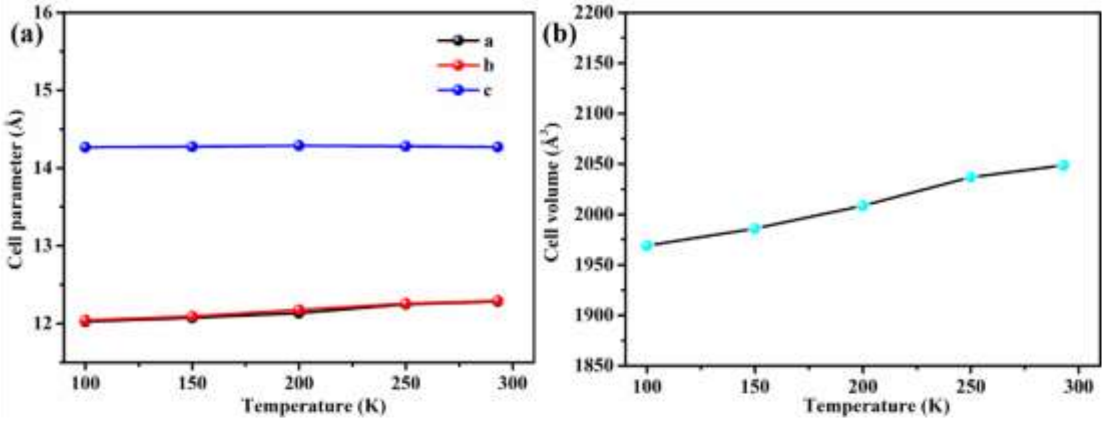

Figure S14. (a) Lattice constants of **Cy_4BiBr_7** as a function of temperature. (b) Cell volume evolution of **Cy_4BiBr_7** based on temperature variations.

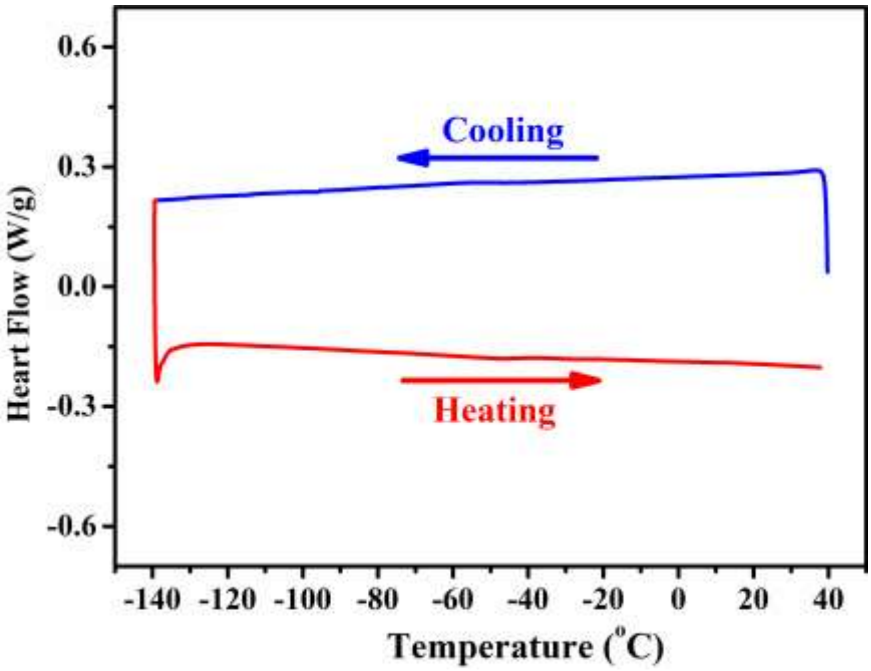

Figure S15. DSC plot for **Cy_4BiBr_7**.

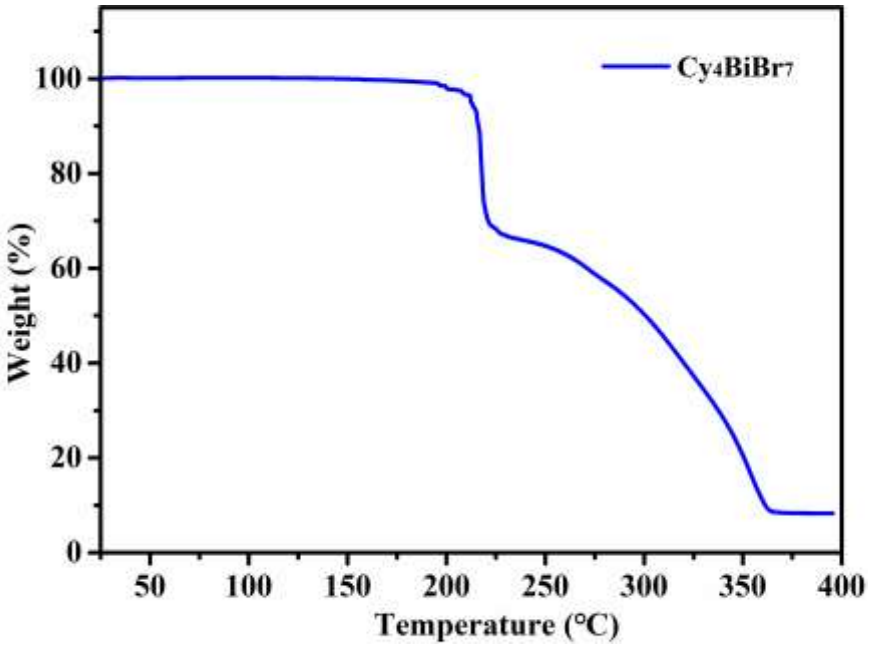

Figure S16. TG curve for $\mathbf{Cy_4BiBr_7}$.

Table S1. Single crystal X-ray diffraction data of **Cy_4BiBr_7** crystal.

Compound	$[(C_6H_{11}NH_3)_4BiBr_6]Br·CH_3CN$ (**Cy_4BiBr_7**)	
Empirical formula	$C_{26}H_{59}BiBr_7N_5$	
Formula weight	1210.13	1210.13
Temperature / K	293(2)	99.97(18)
Crystal system	triclinic	triclinic
Space group	*P*-1	*P*-1
a / Å	12.2824(2)	12.02400(10)
b / Å	12.2937(2)	12.0422(2)
c / Å	14.2700(3)	14.2666(3)
α / °	77.498(2)	103.5080(10)
β / °	77.086(2)	101.0060(10)
γ / °	89.421(2)	90.2510(10)
Volume / $Å^3$	2048.76(7)	1969.05(6)
Z	2	2
ρ_{calc} g / cm^3	1.962	2.041
μ / mm^{-1}	16.609	17.282
F(000)	1156.0	1156.0
Radiation	Cu $K\alpha$ (λ = 1.54184)	Cu $K\alpha$ (λ = 1.54184)
2θ range for data collection / °	6.514 to 129.99	6.5 to 145.25
Index ranges	-14 ≤ h ≤ 10, -13 ≤ k ≤ 14, -16 ≤ l ≤ 16	-13 ≤ h ≤ 14, -14 ≤ k ≤ 14, -17 ≤ l ≤ 17
Reflections collected	19127	20697
Independent reflections	6960 [R_{int} = 0.0507, R_{sigma} = 0.0425]	7547 [R_{int} = 0.0293, R_{sigma} = 0.0270]
Data/restraints/parameters	6960/355/357	7547/0/358
Goodness-of-fit on F^2	1.103	1.107
Final R indexes [I >= 2σ (I)]	R_1 = 0.0728, wR_2 = 0.2208	R_1 = 0.0401, wR_2 = 0.1108
Final R indexes [all data]	R_1 = 0.0752, wR_2 = 0.2245	R_1 = 0.0410, wR_2 = 0.1115
Largest diff. peak/hole / e $Å^{-3}$	4.37 / -3.27	3.67 / -2.25
CCDC NO.	1936596	1974125

$R_1 = \sum||Fo|-|Fc|/\sum||Fo|$. $wR_2 = [\sum w(Fo^2 - Fc^2)^2/\sum w(Fo^2)^2]$

Table S2. Selected bond lengths (Å) and bond angles (°) for **Cy_4BiBr_7**.

Bond lengths				Bond angles			
298 K		100 K		298 K		100 K	
**Bi1-Br1**	**2.8502(10)**	**Bi1-Br1**	**2.8760(7)**	**Br1-Bi1-Br2**	**88.48(4)**	**Br1-Bi1-Br2**	**87.04(2)**
**Bi1-Br2**	**2.8529(10)**	**Bi1-Br2**	**2.8364(8)**	**Br1-Bi1-Br3**	**90.12(3)**	**Br1-Bi1-Br3**	**92.05(2)**
**Bi1-Br3**	**2.8506(9)**	**Bi1-Br3**	**2.8413(7)**	**Br1-Bi1-Br4**	**90.19(4)**	**Br1-Bi1-Br4**	**91.22(2)**
**Bi1-Br4**	**2.8568(10)**	**Bi1-Br4**	**2.8650(8)**	**Br1-Bi1-Br5**	**90.97(3)**	**Br1-Bi1-Br5**	**90.31(2)**
**Bi1-Br5**	**2.8615(9)**	**Bi1-Br5**	**2.8565(7)**	**Br1-Bi1-Br6**	**178.46(3)**	**Br1-Bi1-Br6**	**173.52(2)**
**Bi1-Br6**	**2.8471(11)**	**Bi1-Br6**	**2.8509(7)**	**Br2-Bi1-Br3**	**90.05(3)**	**Br2-Bi1-Br3**	**91.84(2)**
				**Br2-Bi1-Br4**	**178.52(2)**	**Br2-Bi1-Br4**	**177.93(2)**
				**Br2-Bi1-Br5**	**90.22(3)**	**Br2-Bi1-Br5**	**90.43(2)**
				**Br2-Bi1-Br6**	**90.05(4)**	**Br2-Bi1-Br6**	**87.67(2)**
				**Br3-Bi1-Br4**	**90.61(3)**	**Br3-Bi1-Br4**	**89.34(2)**
				**Br3-Bi1-Br5**	**178.89(2)**	**Br3-Bi1-Br5**	**176.80(2)**
				**Br3-Bi1-Br6**	**89.40(4)**	**Br3-Bi1-Br6**	**91.86(2)**
				**Br4-Bi1-Br5**	**89.14(3)**	**Br4-Bi1-Br5**	**88.46(2)**
				**Br4-Bi1-Br6**	**91.28(4)**	**Br4-Bi1-Br6**	**93.99(2)**
				**Bi5-Bi1-Bi16**	**89.52(4)**	**Bi5-Bi1-Bi16**	**85.99(2)**

Table S3. Parameters of the hydrogen bonds in $\mathbf{Cy_4BiBr_7}$ at RT.

D-H	d(D-H)	d(H..A)	<DHA	d(D..A)	A
N1-H1A	0.89	2.969	130.99	3.615	Br4
N1-H1A	0.89	2.846	124.69	3.432	Br4[1]
N1-H1B	0.89	2.529	169.58	3.408	Br7
N1-H1C	0.89	3.022	116.57	3.512	Br5[1]
N1-H1C	0.89	2.882	157.53	3.72	Br6
N2-H2A	0.89	2.946	120.37	3.482	Br3
N2-H2A	0.89	2.769	139.12	3.491	Br3[2]
N2-H2B	0.89	2.567	155.98	3.4	Br7
N2-H2C	0.89	2.767	172.2	3.651	Br1
N3-H3A	0.89	2.795	135.86	3.489	Br2[3]
N3-H3A	0.89	2.897	123.41	3.468	Br2[2]
N3-H3B	0.89	2.514	167.85	3.39	Br7
N3-H3C	0.89	2.782	166.95	3.654	Br6[2]
N4-H4A	0.89	2.791	172.24	3.675	Br1[4]
N4-H4B	0.89	2.534	161.84	3.391	Br7[5]
N4-H4C	0.89	2.85	131.02	3.499	Br5[6]
N4-H4C	0.89	2.856	127.97	3.475	Br5[4]
C6-H6A	0.97	3.114	127.72	3.786	Br1
C8-H8A	0.97	3.14	136.16	3.898	Br7
C26-H26A	0.96	3.111	128.86	3.788	Br5[7]
C26-H26B	0.96	2.823	160.85	3.743	Br3
C26-H26C	0.96	2.816	163.42	3.746	Br2[8]

Table S4. Parameters of the hydrogen bonds in **Cy_4BiBr_7** at 100 K.

D-H	d(D-H)	d(H..A)	<DHA	d(D..A)	A
N1-H1A	0.89	3.1	119.49	3.621	Br4
N1-H1A	0.89	2.626	139.09	3.349	$Br4^{9}$
N1-H1B	0.89	2.548	163.15	3.41	Br7
N1-H1C	0.89	2.938	119.13	3.46	$Br5^{9}$
N1-H1C	0.89	2.773	147.55	3.556	$Br6^{9}$
N2-H2A	0.89	3.012	111.04	3.433	Br4
N2-H2A	0.89	2.711	154.28	3.534	Br1
N2-H2B	0.89	2.736	136.48	3.437	Br3
N2-H2B	0.89	3.107	123.3	3.671	$Br3^{10}$
N2-H2C	0.89	2.515	168.12	3.391	Br7
N3-H3A	0.89	2.659	134.17	3.341	$Br2^{11}$
N3-H3A	0.89	2.865	126.52	3.469	$Br2^{10}$
N3-H3B	0.89	2.557	161.07	3.411	Br7
N3-H3C	0.89	2.655	176.79	3.544	$Br6^{10}$
N4-H4A	0.89	2.658	136.81	3.362	$Br5^{12}$
N4-H4A	0.89	2.854	124.83	3.441	$Br5^{13}$
N4-H4B	0.89	2.661	175.07	3.549	$Br1^{13}$
N4-H4C	0.89	2.504	160.17	3.355	$Br7^{14}$
C8-H8B	0.97	3.075	142.6	3.891	Br7
C20-H20B	0.97	3.078	136.91	3.844	$Br7^{14}$
C26-H26A	0.96	2.833	151.81	3.707	$Br2^{15}$
C26-H26B	0.96	2.791	155.56	3.687	$Br5^{7}$
C26-H26C	0.96	3.073	120.81	3.659	$Br4^{10}$

Symmetry codes: ^{1} *-x, -y-1, -z-1, -z + 1;* ^{2} *-x, -y-2, -z-1;* ^{3} *x+1, y, z;* ^{4} *-x-1, -y-2, -z;* ^{5} *-x,-y-2,-z;* ^{6} *x, y-1, z+1;* ^{7} *x, y-1, z;* ^{8} *-x-1, -y-2, -z-1;* ^{9} *-x+1, -y+3, -z+1;* ^{10} *-x+1, -y+2, -z+1;* ^{11} *x-1, y, z;* ^{12} *x-1, y-1, z-1;* ^{13} *-x+1, -y+2, -z;* ^{14} *-x, -y+2, -z;* ^{15} *-x+2, -y+2, -z+1.*

Table S5. Lattice constants of **Cy_4BiBr_7** under pressure obtained from the refinement of ADXRD patterns.

**Pressure (GPa)**	***a*** **/ Å**	***b*** **/ Å**	***c*** **/ Å**	***α*** **/ °**	***β*** **/ °**	***γ*** **/ °**	**Volume / $Å^3$**
0	11.9622	13.1929	14.9875	64.7615	92.7117	97.8769	2119.14
0.71	11.7069	13.1679	14.5478	64.2424	94.9296	97.9709	1999.46
1.53	11.6812	13.0529	14.2216	64.4452	95.8257	97.9428	1935.39
2.61	11.4719	12.7431	13.9188	64.6247	96.2864	98.0685	1817.48
3.09	11.4521	12.7363	13.8883	64.3802	96.0685	97.7272	1807.4
4	11.4176	12.7931	13.7719	64.1647	96.557	98.0112	1789.65
4.64	11.3788	12.6874	13.7238	63.668	97.1967	98.6139	1751.79
5.84	11.3395	12.6401	13.6407	63.3196	97.7523	98.8062	1721.53
7.98	11.0604	12.6307	13.5665	62.217	97.8478	99.3534	1650.46
8.9	10.9944	12.6076	13.6266	61.1698	98.0214	99.702	1627.31
10.25	10.9445	12.5939	13.6123	60.7024	97.7728	99.7221	1609.72
11.02	10.9342	12.4793	13.573	60.9621	97.3967	99.2153	1595.56
12.28	10.9063	12.4419	13.5134	60.8552	97.3488	99.2084	1578.24
13.94	10.8608	12.4306	13.4587	60.9184	97.1689	99.0823	1565.59
15.8	10.8493	12.3949	13.4423	60.7756	97.245	99.3197	1554.43
17.91	10.8074	12.3707	13.4386	60.8025	97.4827	99.4239	1544.59
20.13	10.7498	12.3319	13.395	61.2461	97.1461	99.1755	1534.51

Table S6. Summary of bond length distortions, angle variance of 0D metal halide octahedron reported in the literature.

Compound	Δd (*10^{-4})	δ^2	Ref.
$(C_4H_{14}N_2)_2In_2Br_{10}$	1.1	1.1	S6
$(C_6H_5CH_2NH_3)_3InBr_6$	1.1	4.99	S8
$(C_6H_5CH_2NH_3)_3SbBr_6$	7.1	14.60	S9
$(C_4N_2H_{14}Br)_4SnBr_6$	45.69	18.20	S10
$(C_4N_2H_{14}Br)_4SnI_6$	1.57	19.45	S10
$(C_3N_3H_{11}O)_2PbBr_6{\cdot}4H_2O$	0.00438	2.57	S11
$(C_8NH_{12})_4BiBr_7{\cdot}H_2O$	12.34	10.62	S12
$(C_6H_5CH_2NH_3)_3BiBr_6$	2.1	14.57	S9
$[(CH_3)_2NH_2][C_6H_5CH_2NH_3]_2BiBr_6$	2.6	7.35	S13
$[(C_6H_{11}NH_3)_4BiBr_6]Br{\cdot}CH_3CN$ (RT)	0.0276	0.61	This work
$[(C_6H_{11}NH_3)_4BiBr_6]Br{\cdot}CH_3CN$ (100 k)	0.245	5.62	This work

References

[S1] CrysAlisPro 2012, Agilent Technologies. Version 1.171.36.31.

[S2] Sheldrick, G. M. *Acta Cryst. A,* **2015**, 71, 3-8.

[S3] Sheldrick, G. M. *Acta Cryst. A,* **2008**, 64, 112-122.

[S4] Dolomanov, O. V.; Bourhis, L. J.; Gildea, R. J.; Howard, J. A. K.; Puschmann, *H. J. Appl. Cryst*., **2009**, 42, 339-341.

[S5] Brandenburg, K. *Diamond*, 2010.

[S6] Zhou, L.; Liao, J. F.; Huang, Z. G.; Wei, J. H.; Wang, X. D.; Chen, H. Y.; Kuang, D. B., *Angew. Chem. Int. Ed. Engl.*, **2019,** *58* (43), 15435-15440.

[S7] Lin, H.; Zhou, C.; Chaaban, M.; Xu, L.-J.; Zhou, Y.; Neu, J.; Worku, M.; Berkwits, E.; He, Q.; Lee, S.; Lin, X.; Siegrist, T.; Du, M.-H.; Ma, B., *ACS Materials Lett.,* **2019,** *1* (6), 594-598.

[S8] D. Chen, S. Hao, G. Zhou, C. Deng, Q. Liu, S. Ma, C. Wolverton, J. Zhao, Z. Xia, *Inorg. Chem.,* **2019**, *58,* 15602-15609.

[S9] D. Chen, F. Dai, S. Hao, G. Zhou, Q. Liu, C. Wolverton, J. Zhao, Z. Xia, *J. Mater. Chem. C,* **2020**, *8*, 7322-7329.

[S10] C. Zhou, H. Lin, Y. Tian, Z. Yuan, R. Clark, B. Chen, L. J. van de Burgt, J. C. Wang, Y. Zhou, K. Hanson, Q. J. Meisner, J. Neu, T. Besara, T. Siegrist, E. Lambers, P. Djurovich, B. Ma,·*Chem. Sci.,* **2018**, *9,* 586-593.

[S11] B. B. Cui, Y. Han, B. Huang, Y. Zhao, X. Wu, L. Liu, G. Cao, Q. Du, N. Liu, W. Zou, M. Sun, L. Wang, X. Liu, J. Wang, H. Zhou, Q. Chen, *Nat. Commun.*, **2019**, *10,* 5190.

[S12] R. Zhang, X. Mao, Y. Yang, S. Yang, W. Zhao, T. Wumaier, D. Wei, W. Deng, K. Han, *Angew. Chem. Int. Ed. Engl.,* **2019**, *58*, 2725-2729.

[S13] B. Wang, D. Ma, H. Zhao, L. Long, L. Zheng, *Inorg. Chem.*, **2019**, *58*, 13953-13959.
